# Influence of deoxynivalenol and zearalenone on the immunohistochemical expression of oestrogen receptors and liver enzyme genes in vivo in prepubertal gilts

**DOI:** 10.1007/s00204-023-03502-7

**Published:** 2023-06-16

**Authors:** Magdalena Gajęcka, Iwona Otrocka-Domagała, Paweł Brzuzan, Łukasz Zielonka, Michał Dąbrowski, Maciej T. Gajęcki

**Affiliations:** 1 Department of Veterinary Prevention and Feed Hygiene, Faculty of Veterinary Medicine, University of Warmia and Mazury in Olsztyn, Oczapowskiego 13/29, 10-718, Olsztyn, Poland; 2 Department of Pathological Anatomy, Faculty of Veterinary Medicine, University of Warmia and Mazury in Olsztyn, Oczapowskiego 13D, 10-718, Olsztyn, Poland; 3 Department of Environmental Biotechnology, Faculty of Environmental Sciences and Fisheries, University of Warmia and Mazury in Olsztyn, Słoneczna 45G, 10-719, Olsztyn, Poland

**Keywords:** Deoxynivalenol, Zearalenone, Liver, Immunohistochemistry ERs, mRNA liver enzymes, Pre-pubertal gilts

## Abstract

Deoxynivalenol (DON) and zearalenone (ZEN) are often detected in plant materials used to produce feed for pre-pubertal gilts. Daily exposure to small amounts of these mycotoxins causes subclinical conditions in pigs and affects various biological processes (e.g. mycotoxin biotransformation). The aim of this preclinical study was to evaluate the effect of low monotonic doses of DON and ZEN (12 µg/kg body weight—BW—and 40 µg/kg BW, respectively), administered alone or in combination to 36 prepubertal gilts for 42 days, on the degree of immunohistochemical expression of oestrogen receptors (ERs) in the liver and the *m*RNA expression of genes encoding selected liver enzymes during biotransformation processes. The level of expression of the analysed genes proves that the tested mycotoxins exhibit variable biological activity at different stages of biotransformation. The biological activity of low doses of mycotoxins determines their metabolic activity. Therefore, taking into account the impact of low doses of mycotoxins on energy-intensive processes and their endogenous metabolism, it seems that the observed situation may lead to the activation of adaptation mechanisms.

## Introduction

Mycotoxins are secondary fungal metabolites which pose a significant threat for global food and feed security due to their adverse effects on human and animal health (Viegas et al. 2019), high chemical stability and ubiquitous presence (Zhou et al. 2017). Simultaneous exposure to several mycotoxins produced by the same or different fungal species exacerbates the risk of food and feed toxicity (Knutsen et al. 2017; Payros et al. 2016). According to research, plant materials are often contaminated with both DON and ZEN, and the health risks associated with simultaneous exposure to both mycotoxins constitute an interesting topic of study (Medina et al. 2017; Zachariasova et al. 2014).

Present in plant material, DON and ZEN belong to a large group of structurally related sesquiterpenoids which are produced by various fungal species, including *Fusarium*, *Myrothecium*, *Cephalosporium*, *Verticimonosporium* and *Stachybotrys* (Zhou et al. 2017). To date, the following mechanisms of toxicity of this mycotoxins have been identified in cells or proteins: (i) DON binds to the 60S ribosome subunit at the molecular level and induces ribotoxic stress which activates protein kinase and, consequently, inhibits protein synthesis, provokes endoplasmic reticulum stress (You et al. 2021), cell signalling, cell differentiation, cell proliferation and cell death (Gajęcka et al. 2021; Pavros et al. 2016); (ii) ZEN exerts toxic effects by binding to and activating both ERs, disrupting the cell cycle and inducing DNA fragmentation, which leads to the production of micronuclei and chromosomal aberrations (Gajęcka et al. 2020; Knutsen et al. 2017; Payros et al. 2016; Shanle and Xu 2010).

The physiological functions of endogenous and exogenous oestrogens are modulated mainly by two subtypes of ERs: ER*α* and Erβ (Paterni et al. 2014). These receptors are present in the cell nucleus where they regulate the transcription of target genes by binding to DNA regulatory sequences. Both subtypes of ERs are expressed in numerous cells and tissues where they regulate specific processes (Gruber-Dorninger et al. 2023; Knutsen et al. 2017). ER*α* is found in various tissues of the reproductive system, bones, adipose tissues and liver where it controls lipid deposition (Chen and Madak-Erdogan 2018; Yasrebi et al. 2017). ER*β* occurs mainly in the prostate epithelium, urinary bladder, ovarian granulosa cells, colon, adipose tissue, immune system and liver, and it is responsible for regulating glucose and lipid metabolism (Chen and Madak-Erdogan 2018; Paterni et al. 2014).

The body maintains homeostasis, therefore all waste products have to be excreted. When this state of equilibrium is disrupted, various compounds are accumulated in the body and may reach toxic levels. Most mycotoxins are biochemically converted to compounds that are more readily soluble in water and can be removed from the body by the liver and kidneys (Piotrowska-Kempisty et al. 2017). This biotransformation process eliminates toxic substances from the body, but it can also contribute to the formation of active metabolites and toxic compounds.

Undesirable substances such as mycotoxins are metabolized inside cells by two classes of enzymes. Phase I enzymes modify undesirable substances via several processes, including hydroxylation. These enzymes are known as cytochromes (CYPs) (Shimizu et al. 2021), they are abundant in the body and tissue-specific. Phase II enzymes, such as glutathione S-transferase (GST) conjugate metabolites through glucuronidation (Cui et al. 2020; Sevior et al. 2012).

The P450 cytochrome (CYP) superfamily consists of several hundred isoenzymes that catalyse the oxidation of various substrates, including exogenous (xenobiotics) and endogenous (hormones, prostaglandins and vitamins) compounds (Goh et al. 2021). Many CYPs are inducible, which significantly increases their catalytic activity after exposure to specific chemical substances (Piotrowska-Kempisty et al. 2017). These compounds are ligands of specific receptors, such as the aryl hydrocarbon receptor (AhR) and ERs. Activated receptors are transferred to the nucleus, they undergo dimerization with nuclear partners, bind to specific sequences in subsequent promotors and induce the transcription of target genes (Freedland et al. 2017). The above increases* m*RNA levels and enhances the synthesis of CYP protein. This process ultimately boosts the enzymatic activity of specific CYPs (Billat et al. 2017).

In phase II, liver cells could become resistant to various substances due to the intensification of metabolic process and detoxification of undesirable compounds in feed (Basharat and Yasmin 2017). The π isoform of glutathione S-transferase (GSTπ1) is one of the molecules that elicit these types of mechanisms (Cui et al. 2020). In the body, GST occurs in the form of numerous isoenzymes which have been divided into classes based on their location in the cell, amino acid sequences, location of genes and substrate specificity (Singh et al. 2017). The role of GSTπ1 is not limited to the detoxification of exogenous electrophilic toxins. The enzyme also protects the body against the harmful products of oxidative stress, and prevents damage to nucleic acids and lipids. Glutathione S-transferase participates in the metabolism of steroid hormones, biosynthesis of leukotriene C4 and prostaglandin E2, and the maintenance of glutathione homeostasis (Kovacevic et al. 2017).

The liver is the body's largest internal organ and plays a key role in controlling energy (Tanaka et al. 2017) and hormonal (Gajęcka et al. 2020) homeostasis by metabolizing nutrients, undesirables substances and/or oestrogen-like substances. Numerous attempts have been made to identify mycotoxin ligands that act as agonists or antagonists (Zanger and Schwab 2013). There is no clear answer. There is also no answer whether during the biotransformation of the discussed mycotoxins there is a change in the expression of ERs genes in the liver and liver enzymes such as CYP and GSTπ1.

Therefore, the aim of this study was to determine whether a low monotonic dose of DON and ZEN, applied *per os*, in vivo, alone or in combination, affects the immunohistochemical expression of ER*α* and ER*ß* in the liver and the *m*RNA expression of genes encoding selected liver enzymes during biotransformation processes in maturing gilts.

## Materials and methods

The experiment was performed at the Department of Veterinary Prevention and Feed Hygiene, Faculty of Veterinary Medicine, University of Warmia and Mazury in Olsztyn, Poland, on 36 clinically healthy gilts with initial body weight of 25 ± 2 kg. Gilts were penned in groups with ad libitum access to water. Body weight gains in the studied population were described by Gajęcka et al. (2017).

The administered feed was tested for the presence of mycotoxins: ZEN, α-ZEL (α-zearalenol) and DON. Mycotoxin levels were estimated by common separation techniques with the use of immunoaffinity columns (DON-Test^™^ DON Testing System, VICAM, Watertown, USA; Zearala-Test^™^ Zearalenone Testing System, G1012, VICAM, Watertown, USA) and high-performance liquid chromatography (HPLC) (Hewlett Packard, type 1050 and 1100) (Liu et al. 2021) with fluorescent and/or UV detection techniques. The limit of detection was 1.0 ng/g for DON (Waśkiewicz et al. 2014) and 1.0 ng/g for ZEN (Zielonka et al. 2015).

Experimental design—the animals were divided into DON (n = 9), ZEN (n = 9) and MIX (DON + ZEN, n = 9) experimental groups and a control group (CON, n = 9) (Heberer et al. 2007; Smith et al. 2005). The animals from the experimental groups were orally administered DON at 12 μg/kg BW (group DON), ZEN at 40 μg/kg BW (group ZEN) or both mycotoxins—DON at 12 μg/kg BW + ZEN at 40 μg/kg BW (group MIX = DON + ZEN). Group C pigs were fed a placebo. When the experiment was designed, the above values were consistent with EFSA guidelines (Commission Recommendation 2006) as the so-called NOAEL dose (no-observed-adverse-effect level). Mycotoxins were administered daily in gastro-soluble gel capsules (two-piece gel capsules), half an hour before morning feeding. Feed was the carrier, and C group pigs were administered the same gel capsules, but without mycotoxins.

Both mycotoxins were biosynthesized, purified and standardized by the Department of Chemistry of the Poznań University of Life Sciences, Poznań, Poland. The experiment covered a period of 42 days. Zearalenone and DON doses were adjusted to the BW of the experimental animals. The mycotoxins were administered in capsules to prevent problems associated with uneven feed intake. Mycotoxin samples were diluted in 500 μL of 96% ethyl alcohol (96% ethyl alcohol, SWW 2442-90, Polskie Odczynniki Chemiczne SA, Poland) to the required dose level (based on BW). Final solutions were stored at room temperature for 12 h to evaporate the solvent. The animals were weighed every 7 days to adjust mycotoxin doses for each gilt. Three animals from each of the four groups (experimental and control) were sacrificed on days 7 (date I—DI), 21 (date II—DII), and 42(date III—DIII), (a total of 12 gilts on each date). Every date, twelve animals were euthanized by intravenous administration of pentobarbital sodium (Fatro, Ozzano Emilia BO, Italy) and bleeding. Sections of liver tissues were collected immediately after cardiac arrest and were prepared for analyses. Zearalenone and DON were synthesized and standardized based on a previously developed procedure (Kostecki et al. 1991a, 1991b) presented in other studies (Gajęcka et al. 2020). A chromatographic analysis of DON and ZEN was in accordance with a previously described procedure (Muñoz-Solano and González-Peñas 2020; Wiśniewska et al. 2014).

Tissue samples—every date, tissue samples from the porcine liver were collected post-mortem and rinsed with phosphate buffer. Tissue samples (approximately 1 × 1.5 cm) were cut from the same segment of the left liver lobe within 3 min after cardiac arrest. Tissue samples were collected from entire liver cross-sections, and they were stored at a temperature of − 20 °C. The samples were labelled with a code to prevent identification by researchers who evaluated and described liver tissues.

### Immunohistochemistry

Tissue specimens were fixed in 4% paraformaldehyde solution and embedded in paraffin. Two specimens of every examined liver clippings were stained to determine the expression of ER*α* and ER*β*. The analytical procedure has been described previously (Gajęcka et al. 2021; Singhai et al. 2011).

**Fig. 1:**
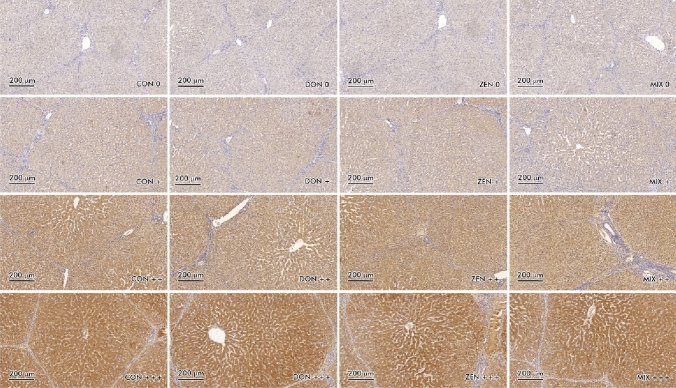
Scanned slides of immunohistochemical expression of ER*α* receptors (brown color) in the liver, presented vertically: in group C (CON 0; CON + ; CON +  + ; CON +  + +), in group DON (DON 0; DON + ; DON +  + ; DON +  + +), in group ZEN (ZEN 0; ZEN + ; ZEN +  + ; ZEN +  + +) and in group MIX (MIX 0; MIX + ; MIX +  + ; MIX +  + +). HE

**Fig. 2:**
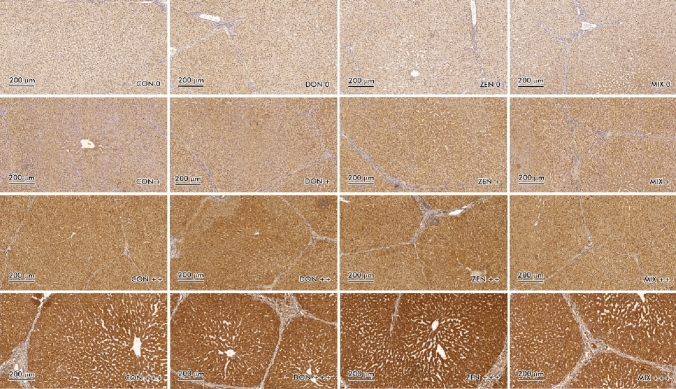
Scanned slides of immunohistochemical expression of ER*ß* receptors (brown color) in the liver, presented vertically: in group C (CON 0; CON + ; CON +  + ; CON +  + +), in group DON (DON 0; DON + ; DON +  + ; DON +  + +), in group ZEN (ZEN 0; ZEN + ; ZEN +  + ; ZEN +  + +) and in group MIX (MIX 0; MIX + ; MIX +  + ; MIX +  + +). HE

Optical density (scanning) of stained slides—the expression of ER*α* and ER*β* in liver samples from both groups was analysed in scanned slides (Pannoramic MIDI Scanner, 3DHISTECH, Budapest, Hungary) using the NuclearQuant program (3DHISTECH, Hungary). Scanned slides were converted into digital images (Figs. 1 and 2). Nuclear immunoreactivity was evaluated. The nucleus detection profile was as follows: radius—1.50–2.10 µm, minimum nuclear area—0.9 µm, minimum circularity—3, smoothness—1. ER*α* and ER*β* expression was evaluated on a 4 point scale: negative = 0 [0], weak and homogeneous =  + [1], mild or moderate and homogeneous =  +  + [2], intense or strong and homogeneous =  +  +  + [3]. Staining intensity was evaluated on the following scale: from 0—none of the below, + average intensity < 190 (CD BrownInt), +  + average intensity < 170 (CD BrownInt), +  +  + average intensity < 100 (CD BrownInt) (Gajęcka et al. 2021). The results were expressed by the average percentage of hepatocytes with ER*α* and ER*β* expression.

### Expression of CYP1A1 and GSTπ1 genes

Collection and storage of samples for RNA extraction—immediately after cardiac arrest, fragments of the liver were collected and stored in RNA*later* solution (Sigma-Aldrich; Germany) according to the manufacturer’s recommendations. Total RNA was extracted from the tissues preserved in RNA*later* (approx. 20 mg per sample; *n* = 3 in each experimental group) using the Total RNA Mini isolation kit (A&A Biotechnology; Poland). The analytical procedure was described earlier (Gajęcka et al. 2021).

Real-Time PCR primers for target mRNAs were designed using the Primer-BLAST tool (Ye et al. 2012) based on reference species (Table 1). The real-time PCR assay was performed in the ABI 7500 Real-Time PCR system thermocycler (Applied Biosystems; USA) in singleplex mode. Further treatments were applied according to the manufacturer’s recommendations. The analytical procedure was described earlier (Gajęcka et al. 2021).

**Table 1:** Real-time PCR primers used in this study

Gene	Primer	Sequence (5′ → 3′)	Amplicon length (bp)	Reference
CYP1A1	Forward	cagagccgcagcagccaccttg	226	(Gajęcka et al. 2021)
	Reverse	ggctcttgcccaaggtcagcac		
GSTπ1	Forward	acctgcttcggattcaccag	178	(Gajęcka et al. 2021)
	Reverse	ctccagccacaaagccctta		
*β-*Actin	Forward	catcaccatcggcaaaga	237	(Tohno et al. 2006)
	Reverse	gcgtagaggtccttcctgatgt		

Quantitative cycle (Cq) values from qPCR were converted into copy numbers using a standard curve plot (Cq versus log copy number) according to the method proposed by Arukwe (2006) and described by Spachmo and Arukwe (2012). The analytical procedure was described earlier (Gajęcka et al. 2021).

### Statistical analysis

The activity of ERα and ERβ and the expression of CYP1A1 and GSTπ1 genes in the porcine hepatocytes were presented based on mean values ( ±) and standard deviation (SD) for each sample. The results were processed in the Statistica program (StatSoft Inc., USA). Group means (DON, ZEN, DON + ZEN and CON) were compared by repeated measures one-way ANOVA based on the applied doses of DON, ZEN and DON + ZEN. If differences were found between groups, different group pairs were identified in Tukey’s post-hoc test. In ANOVA, the values in groups are drawn from populations with normal distribution and identical variance. These assumptions were not met in all cases, therefore, the equality of group means was analysed in the Kruskal–Wallis test of ranks and a multiple comparison test in ANOVA. Non-parametric tests were used to check for differences in samples.

## Results

The concentration of mycotoxins in the analysed feed was not found or was below the sensitivity of the method (VBS). The concentrations of masked mycotoxins were not analysed.

Clinical signs of DON, ZEN or MIX mycotoxicosis were not observed during the experiment. However, changes in specific tissues or cells were frequently observed in histopathological analyses, ultra-structural analyses and metabolic profile analyses of samples collected from the same animals. The results of these analyses were published in different papers (Gajęcka et al. 2017, 2018, 2020, 2021; Piotrowska et al. 2014; Waśkiewicz et al. 2014; Zielonka et al. 2015).

### Immunohistochemistry

Optical density—Brown staining in the scanned slides (Figs. 1 and 2) was not specific, and it could have occurred during non-specific tissue staining analyses examining the expression of ER*α* and ER*ß* in hepatocytes tissues stained with DAB (non-specific light brown staining was observed in most specimens).

The effect of 42-day exposure to DON, ZEN and MIX on the expression of selected ERs was determined in hepatocytes in four groups on the basis of a 4-point rating scale, which was used in the text. (negative—[0]; weak and homogeneous—i.e. one + as [1]; mild or moderate and homogeneous—i.e. one +  + as [2]; intense or strong and homogeneous—i.e. one +  +  + as [3]). Due to the large number of significant and highly significant differences, the results for hepaticytes are presented not only in tabular form.

ER*α* expression at level [0] was excited more intensely in each date in the CON group compared to the ZEN, DON and MIX groups (Fig. 1, Table 2). In the CON group, significant differences in ER*α* expression were found at different absorption levels (strongest in level [0], and at the other levels they were more muted), but absorption was significantly strongly accentuated in dates II and III (especially in the DON and MIX groups). Significant differences in ER*α* expression were also noted at other absorption levels. The obtained values were much lower than those recorded at the absorption level [0]. In the CON group, mean ER*α* expression was highest at the level of staining [0] and increased with subsequent exposure dates.

**Table 2:** Immunohistochemical expression of ER*α* and ER*β* in the control and exposed groups

ER*α*		DI	DII	DIII
Group CON	0	980.99 ± 422.09^ee,@^	337.66 ± 274.35*^,#,@^	1828.77 ± 297.39^ff,##^
	+	51.66 ± 50.08^aa^	115.99 ± 99.93	51.33 ± 12.72 cc^@^
	+ +	128.88 ± 184.31^aa,##,@@^	376.16 ± 168.99^g,@^	93.88 ± 49.28 cc^@@,#^
	+ + +	137.77 ± 236.32^aa,@,##^	525.5 ± 540.93^g,^*^,#^	29.1 ± 36.1 cc^##,@^
Group ZEN	0	1195.44 ± 465.84	1582.16 ± 1438.22	1602.88 ± 944.71
	+	42.88 ± 64.18^a,b,@^	57.31 ± 48.63^b^	70.11 ± 14.3 b^c,@,#^
	+ +	125.11 ± 208.03^a,b,##,@@^	136.49 ± 98.57^b^	149.99 ± 72.72^b,c,@@^
	+ + +	51.64 ± 87.43^a,b,##^	89.35 ± 82.13^b,##^	157.55 ± 150.57^b,c,#^
Group DON	0	1210.11 ± 977.43	1037.62 ± 1121.72	615.66 ± 676.46^ff^
	+	98.44 ± 121.08^a^	112.52 ± 108.41^a^	132.49 ± 144.01
	+ +	332.55 ± 319.79	485.37 ± 402.64^@^	717.99 ± 689.66^@^
	+ + +	444.88 ± 380.89^d^	1624.03 ± 1106.88	3063.16 ± 2042.83^d,ff^
Group MIX	0	1321.99 ± 694.51	889.33 ± 304.05^g^	1705.99 ± 385.23^##^
	+	137.88 ± 231.04^a^	41.83 ± 3.06^b^	347.77 ± 80.74^c,g^
	+ +	350.55 ± 567.05^a^	65.99 ± 7.54^b^	1254.22 ± 186.25^c,gg^
	+ + +	23.33 ± 39.83^aa,##^	13.83 ± 13.9^bb,^*^,##^	947.1 ± 355.61^cc,ff,gg,#^
ER*β*				
Group CON	0	347.16 ± 329.74 h^ll,mm^	27.52 ± 29.12^h^	22.44 ± 22.84^h^
	+	41.33 ± 18.85 h	19.67 ± 14.58^h^	12.1 ± 10.67^h^
	+ +	127.83 ± 13.43 h	120.46 ± 96.47^h^	117.88 ± 102.12^h^
	+ + +	1051.33 ± 436.37*	1459.72 ± 91.52*^,#^	1803.66 ± 88.43**^,#^
Group ZEN	0	447.00 ± 68.98^l^	73.14 ± 68.42	295.5 ± 359.91
	+	58.55 ± 57.54	20.6 ± 22.75	102.33 ± 110.77^ii^
	+ +	164.77 ± 126.92	191.27 ± 174.6	406.16 ± 259.5^j^
	+ + +	565.33 ± 59.22^k^	1965.48 ± 259.53	1334.16 ± 415.86
Group DON	0	229.1 ± 136.5^h,l^	48.63 ± 47.38^h^	98.66 ± 79.67^h^
	+	52.21 ± 36.41^h^	18.66 ± 20.41^h^	38.33 ± 38.18^h^
	+ +	348.89 ± 234.22^h,l^	117.38 ± 106.04^h^	233.49 ± 296.24^h^
	+ + +	1376.66 ± 80.45**	1910.32 ± 436.22	1550.8 ± 430.94*
Group MIX	0	60.5 ± 60.1	25.35 ± 24.96	215.66 ± 216.81
	+	18.33 ± 17.43	6.3 ± 5.81	48.77 ± 55.37
	+ +	87.49 ± 62.45	116.37 ± 125.74	202.99 ± 191.31
	+ + +	1547.16 ± 505.1	1297.31 ± 84.59**^,##^	1889.66 ± 595.8*^,#,@^

Exposure date: DI—exposure day 7; DII—exposure day 21; DIII—exposure day 42. Experience groups: CON—control group; ZEN—group ZEN at 40 μg/kg BW; DON—group DON at 12 μg/kg BW; MIX—group MIX, both mycotoxins—DON at 12 μg/kg BW + ZEN at 40 μg/kg BW. The expression of ER*α* and ER*β* were determined in the liver in four groups based on a 4-point grading. Statistically significant differences **ER*α*** between the degrees of expression in the experimental groups were determined at ^a^, ^b^, ^c^, ^d^, ^e^, ^g^ p ≤ 0.05 and ^aa^, ^bb^, ^cc^, ^ee^, ^ff^, ^gg^ p ≤ 0.01. Statistically significant differences **ER*β*** between the degrees of expression in the experimental groups were determined at ^h^, ^j^, ^k^, ^l^ p ≤ 0.05 and ^ii^, ^ll^, ^mm^ p ≤ 0.01. A noticeable difference between the experimental groups within the specified exposure date were determined at ^*^, ^#^, ^@^p ≤ 0.05 and ^**^, ^##^, ^@@^p ≤ 0.01

^a^Differences between intensity 0 and other intensities in DI-DIII

^b^Differences between 0 and the others in DII

^c^Differences between 0 and the others in DIII

^d^Differences in DON between + and +  +  +

^e^Are the differences between DI and DII

^f^Are the differences between DI and DIII

^g^Are the differences between DII and DIII

^h^Differences between intensity +  +  + and other intensities in DI-DIII

^i^Differences in ZEN between +  +  + and +

^j^Differences in ZEN between +  +  + and +  + in DI

^k^Differences in ZEN between +  +  + and +  + in DII

^l^Are the differences between DI and DII

^m^Are the differences between DII and DIII

^*,**^Notable difference between the ZEN group and the other experience groups

^#,##^Notable difference between the DON group and the other experience groups

^@,@@^Notable difference between the MIX group and the other experience groups

Analysis of immunohistochemical expression of ER*α* documents that it was significantly elevated in DIII in the DON and MIX groups (in [1], [2] and [3] gradients scale) (Fig. 1, Table 2). In the DON group and partly in the MIX group, ER*α* expression at the absorption level [3] was statistically higher than in the other experimental groups. Differences in ER*α* expression at levels [1], [2] and [3] were observed in all groups of experience over time and especially in the DON and MIX groups. As in the CON group, excitation of ER*α* expression + was observed in the ZEN and DON groups at the absorption level [0] in DIII compared to DI, while at the absorption level [3], ERα expression was more excited in the DON group in DII and DIII, and in the MIX group in DIII, compared to the CON and ZEN groups. Gene expression was stronger in the [0] experimental groups only in DI and DII. As mycotoxin exposure continued, statistical differences were more and more frequently observed between experimental groups.

In the CON group, immunohistochemical ER*ß* expression was muted on all gradients scale and in all DI-DIII (Fig. 2, Table 2), as well as in the MIX group. Mean ER*ß* expression values in all experimental groups and at all exposure times, the expression was most strongly expressed (also statistically) at the absorption level [3]. The greatest silencing was found at the absorption level [0], the observed differences were not always significant. Immunohistochemical analysis of ER*ß* expression in the liver compared to ER*α* expression yielded completely different results. In the experimental groups, ER*ß* expression was more excited at the absorption level [3]. Statistical differences between the experimental groups were found at all exposure dates, alternating in DI in the DON and MIX groups, in DII in the ZEN and DON groups, and in DIII in the CON and MIX groups. The above situation can be explained by the fact that in addition to the tested xenobiotics, other factors had to act, such as the ongoing change in oestrogen clearance, biotransformation processes and the formation of metabolites whose biological activity is much longer in the exposed organism, or environmental factors.

There was a clear presence of a trend. In the case of ER*α* (Table 2), all groups have the highest number of negative receptors. Only in the DON group, similar expression values were found on the [0] scale, defined as negative, and on the [3] scale, defined as intense or strong. On the other hand, ER*β* (Table 2) was expressed on a scale equal to [3], determined as intense or strong, in all groups of the experiment and at all exposure times. It could be suggested that the mycotoxins under study, on the one hand, silence the expression of ER*α*, and on the other hand, more abundant expression of ER*β* genes takes place. However, it should be noted that an analogous situation occurs in the CON group.

**Fig. 3:**
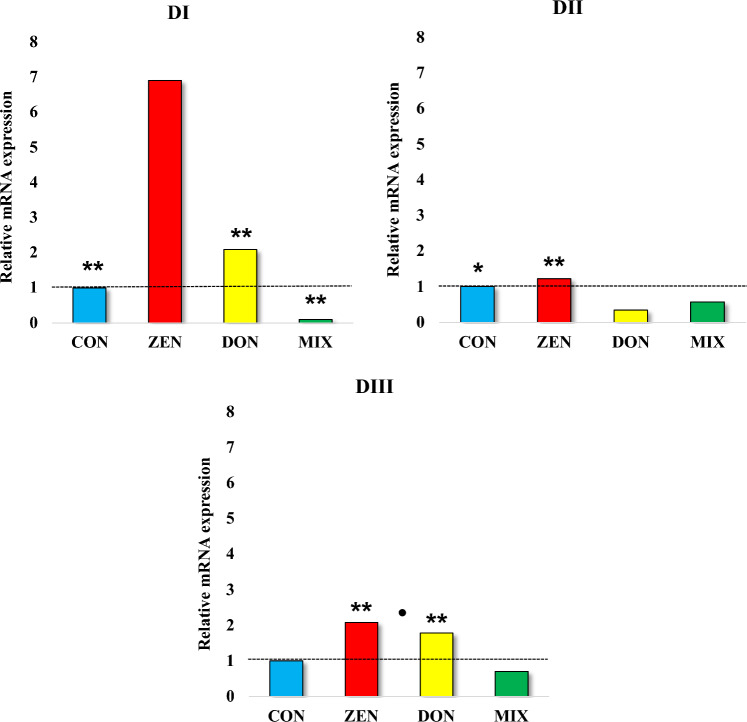
Analytical dates values of CYP1A1 mRNA expression in the livers of pre-pubertal gilts exposed to individual and combined Fusarium mycotoxins. Exposure date: DI—exposure day 7; DII—exposure day 21; DIII—exposure day 42. Experience groups: CON—control group; ZEN—group ZEN at 40 µg/kg BW; DON—group DON at 12 µg/kg BW; MIX—group MIX, both mycotoxins—DON at 12 μg/kg BW + ZEN at 40 μg/kg BW. The expression of CYP1A1 mRNA was determined in the liver in four groups in three dates. The expression was presented as mean values ( ±) and standard deviation (SD) for each sample, relative to the control sample at the beginning of the experiment (ER = 1.00; dashed line). *,^•^p ≤ 0.05 and **p ≤ 0.01 compared with the remaining groups. On DI ** statistical differences between the ZEN group and the CON, DON and MIX groups. On DII *, ** statistical differences between the MIX group and the CON and DON groups, respectively. On DIII **statistical differences between the MIX group and the ZEN and DON groups; ^•^differences between the ZEN group and the DON group

Comparing the results of the research with the hypothesis presented for the purpose of the work, certain statements come to mind. One of them is such that in the end there is a silencing of the expression of the *CYP1A1* gene in the CON and MIX groups. In the other two experimental groups, and especially in the ZEN group, silence also takes place, but at much higher values (Fig. 3). This means that there is a large physiological silence in the case of the CON group, and in the ZEN group this state is much more strongly expressed. This takes place at all dates of liver sampling. Also, silencing the expression of the *CYP1A1* gene in the other two experimental groups may be the result of the biotransformation of a given undesirable substance, i.e. the result of the presence of an independent variable in the experimental groups and its absence in the CON group.

Other dependent substances, such as Glutathione S-Transferases (GST), perform a detoxification function consisting primarily in catalysing the coupling reaction of endogenous glutathione with electrophilic metabolites formed in the first phase of the biotransformation process. These enzymes protect cells both from the harmful effects of chemical compounds with electrophilic properties and from the products of oxidative stress GST. The family of these dimeric enzymes is responsible for the coupling of exogenous and endogenous substances with glutathione, preventing DNA damage by binding toxic compounds in the cytoplasm and thus preventing their interaction with the nucleic acid.

The date expression values of the *GSTπ1* gene differed significantly in groups CON and DON. A comparison of date values between groups revealed significant differences in both enzymes in each date of exposure. The highest values were obtained in the ZEN group at all exposure dates (1.65, 1.74 and 1.45, respectively) and in the DON group at DII (1.89). The lowest expression values were found in the MIX group at all exposure dates (1.08, 1.00 and 0.99, respectively), even compared to the CON group.

**Fig. 4:**
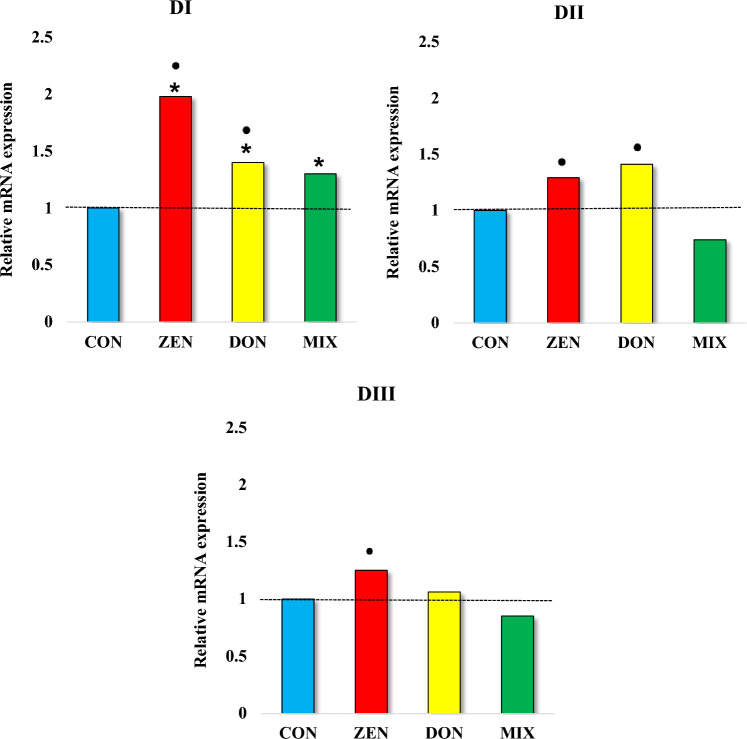
Analytical dates values of *GSTπ1* mRNA expression in the livers of pre-pubertal gilts exposed to individual and combined Fusarium mycotoxins. Exposure date: DI—exposure day 7; DII—exposure day 21; DIII—exposure day 42. Experience groups: CON—control group; ZEN—group ZEN at 40 µg/kg BW; DON—group DON at 12 µg/kg BW; MIX—group MIX, both mycotoxins—DON at 12 μg/kg BW + ZEN at 40 μg/kg BW. The expression of *GSTπ1* mRNA was determined in the liver in four groups in three dates. The expression was presented as mean values ( ±) and standard deviation (SD) for each sample, relative to the control sample at the beginning of the experiment (ER = 1.00; dashed line). *^•^ p ≤ 0.05 compared with the remaining groups. On DI *statistical differences between the CON group and the ZEN, DON and MIX groups; ^•^statistical differences between the MIX group and the ZEN and DON groups. On DII ^•^ statistical differences between the MIX group and the ZEN and DON groups. On DIII ^•^ statistical differences between the MIX group and the ZEN group

Expression of *CYP1A1* and *GSTπ1* genes—Significant differences in the date expression values of the *CYP1A1* gene were noted in all groups (Figs. 3 and 4). This significant number of statistical differences would indicate a large variability in the experimental groups at particular exposure dates, especially at the DI and the ZEN group (1.57). The process of (turbulent) high involvement of the liver in detoxification processes proves that despite the low-dose exposure to mycotoxins, the processes take place in different experimental groups with different intensity and that there is a fairly quick adaptation to the situation (mycotoxicosis), i.e. silencing—especially in DIII in the group MIX (0.35), lower than the CON group, which is consistent with the thoughts presented by Schmidhauser et al. (2023).

## Discussion

Physiologically (as is the case in group CON), the level of expression of ERs proteins in maturing gilts is very different depending on the degree of sexual maturity (Schmidhauser et al. 2023). This is due to the fact that ERs are mediators of oestrogens or substances with oestrogen-like activity or oestrogen disrupter, such as ZEN (Gajęcka et al. 2023; Rykaczewska et al. 2019) or also even DON (Gajęcka et al. 2021). In the situation of physiological deficiency of endogenous oestrogens (during puberty), there is an increased readiness, mainly ER*β* (at [3] gradients scale) and ER*α* are basically inactive, which was confirmed by the presented results (Table 2). Oestrogen alpha receptors inhibit the activity of steroid hormones that convert circulating hormones into E_2_ (Rykaczewska et al. 2019), which causes a delay in the sexual maturation of female organisms. In turn, ER*β* are responsible for intensified to speed up metabolic processes (Savva and Korach-André 2020) or maturation of the body (Gajęcka et al. 2023; Yoon et al. 2020).

Oestrogen receptors alpha and beta (ER*α* and ER*β*) are type 3 nuclear receptors (Tanaka et al. 2017) which participate in the regulation of complex physiological processes (Galuszka et al. 2021) including hormonal homeostasis. An analysis of the modulatory effects exerted by undesirable substances on these receptors contributes to our understanding of the aetiology of various states, including non-pathological states. In the described experiment, the applied mycotoxin doses were low, therefore, our results cannot be compared with the findings of other authors.

The percentage of negative stains was highest in CON and MIX groups, but their distribution was not uniform. In the ZEN group, the percentage of negative stains in successive weeks was high and stable (Table 2). The strongest expression (3 points) denoting the most intense staining was noted only in the DON group where immunoreactivity increased gradually over time (Gajęcka et al. 2020, 2021). Despite the above, the mechanism by which a non-oestrogenic compound can act as an ERs ligand remains unknown (Gajęcka et al. 2021).

The immunoreactivity of ER*α* corresponding to 1, 2 and 3 points on the grading scale (stain intensity) was generally very low (nearly negative). Staining intensity in ZEN and MIX groups (i.e. ZEN administered individually and in combination with DON) was similar to that observed in the CON group. The results noted in ZEN and MIX groups are consistent with the findings of Wang and colleagues (Wang et al. 2015) who observed that higher ER*α* immunoreactivity in the liver plays a key role in inhibiting triglyceride synthesis and cumulation. Our results suggest that these processes are particularly important for energy cumulation in the livers of pre-pubertal animals (Gajęcka et al. 2017). The highest percentage of negative stains (0 points) was observed in both experimental groups. Similar results were reported by other authors (Tanaka et al. 2017). Other researchers have suggested the ZEN (mycoestrogen) could control ER*α* (Besse-Patin et al. 2017; Gajęcka et al. 2018). The only exception was noted in the DON group where ER*α* immunoreactivity was strong (3 points). This observation is very difficult to interpret because the relevant mechanisms have not yet been elucidated. In previous own studies, but at the intestinal level, similar immunoreactivity of ER*α* was noted (Gajęcka et al. 2021). Returning to the assessed results, the data presented in Table 2 also indicate that the expression of ER*α* (3 points) in the DON group increased in the DII and DIII date of the study. We believe that this is not a methodological error because the results of replicate analyses produced comparable results. The most significant differences in expression were observed between successive dates of the experiment, in particular in CON and MIX groups. Expression levels were most stable in the ZEN group, which suggests that this mycoestrogen could have a stabilizing influence on ER*α* expression.

It should also be noted that mycotoxins have a bactericidal and/or bacteriostatic effect and are able to modify the intestinal microbiota (Di Domenico et al. 2022; Piotrowska et al. 2014; Reddy et al. 2018). Alternative gut microbiota can synthesize other by-products which act as ERs ligands and participate in metabolic processes (Chen and Madak-Erdogan 2018).

The analysed ER*α* ligands differed in immunoreactivity. In animals administered ZEN alone and ZEN + DON, the cumulation of feed-derived energy was inhibited, and similar results were observed in the CON group. The results noted in the DON group are difficult to interpret, and they could suggest that the analysed mycotoxin intensified the decomposition of energy compounds (Gajęcka et al. 2021).

In all groups, the immunoreactivity of ER*ß* was strong (3 points) in the majority of cases (Table 2). However, a steady and proportional increase in ER*ß* expression approximating the physiological norm (Božovićet al. 2021; Savva and Korach-André 2020) was observed only in the CON group (Paterni et al. 2014). In the ZEN group, the immunoreactivity of ER*ß* was also strong (3 points) in the majority of cases, but it was considerably lower than in the remaining groups. In the third date of exposure, ER*ß* expression (3 points) decreased in all groups, and the greatest decrease was noted in the ZEN group. The presented situation is confirmed by the results presented by Nagl and colleagues (2021), where the intensification of metabolic processes during zearalenone mycotoxicosis was documented. Pre-pubertal females are characterised by low levels of endogenous oestrogens; therefore, the presence of ZEN in feed (administered alone and in combination with DON) could weaken ER*ß* expression (3 points) (Table 2) relative to the remaining groups (Gruber-Dorninger et al. 2023; Zheng et al. 2019). At the same time, this condition contributes to the slowing down of the maturation processes of prepubertal gilts and the deposition of spare substances (Nagl et al. 2021).

The results of ERs analysis suggest that some undesirable substances could increase ERs expression to the two highest levels on the applied grading scale in the last two dates of exposure. The expression of ER*α* was intensified to cumulate energy in liver cells (DON and MIX groups) (Wang et al. 2015); whereas the expression of ER*ß* was intensified to speed up metabolic processes (Gajęcka et al. 2017; Savva and Korach-André 2020) or maturation (Robert et al. 2017; Yoon et al. 2020). Our results corroborate the findings of Chen and Madak-Erdogan (2018) who observed that the activation of both ER*α* and ER*ß* may contribute to metabolic regulation in hepatocytes.

To sum up, this could be considered a two-way action—on the one hand, there are endogenous oestrogens and xenoestrogens, and on the other hand, it is a stage of the maturation process of the female body. The coexistence of these three factors has different effects on the degree of expression of ERs. This allows the body to have high developmental plasticity, which in turn allows it to adapt to specific environmental conditions in order to achieve the greatest chances of survival and reproductive success (Schmidhauser et al. 2023).

Hepatic biotransformation converts biologically active xenoestrogens and endogenous oestrogens into hydrophilic metabolites, thus preventing binding to ERs and facilitating their excretion in bile and urine. Enzymatic sulphation and glucuronidation processes as a result of conjugation are the basic stages of elimination of steroidogenic compounds. Undesirable substances, including mycotoxins (ZEN and DON), are metabolized inside cells, mainly in hepatocytes, by two classes of enzymes. The activity of enzymes participating in these processes is determined by various physiological and pathological factors (Billat et al. 2017; Yoon et al. 2020). The present study makes a pioneering attempt to determine the influence of low mycotoxin doses on the expression of genes encoding selected liver enzymes, which is why our results are difficult to compare with the findings of other authors.

In the first date of the exposure, the *m*RNA expression of *CYP1A1* was higher in all experimental groups than in the CON group (except for the MIX group). In the first two dates of exposure, the *m*RNA expression of *CYP1A1* increased only in the CON group (Fig. 3—DII and DIII). The above can probably be attributed to the fact that CYP1A1 is responsible for the biotransformation of undesirable compounds (Li et al. 2016) through the hydroxylation of oestrogens or oestrogen-like substances (Piotrowska-Kempisty et al. 2017). Therefore, the presence of mycotoxins, in particular ZEN, in feed could increase the expression of the *CYP1A1* gene (Goh et al. 2021). In the remaining weeks of exposure, the *m*RNA expression of *CYP1A1* was lower in the MIX group than in the CON group. The analysed gene was always more strongly expressed in the ZEN group, by 50% on average, than in the remaining groups (Fig. 3 DI). These findings suggest that ZEN (administered individually and in combination with DON), probably as a substrate not an inhibitor, enhances expression the *m*RNA of *CYP1A1* (Freedland et al. 2017; Nguyen et al. 2022; Pan et al. 2023). Previous research findings (Gajęcka et al. 2020, 2021) postulating that ZEN could inhibit the *m*RNA expression of *CYP*_*SCC*_ were not confirmed. The main differences were the mycotoxin dose (50 and 75 μg ZEN/kg BW), tissue sampling sites (ovaries) and animal species. The present findings suggest that low doses of oestrogen-like substances enhance the *m*RNA expression of *CYP1A1* in pre-pubertal gilts, which is consistent with the “low dose” theory (Vandenberg et al. 2012).

Contrary results were observed in the experimental groups where the *m*RNA expression of *CYP1A1* was less inhibited under exposure to DON alone than under exposure to both mycotoxins. A decrease in the expression of the analysed gene could point to the loss of specialised liver functions (Smith et al. 2017) or the fact that DON was not biologically active in the first stage of biotransformation (Payros et al. 2016). The above could suggest that the biological activity of parent compounds is related to their metabolic activity (Shimizu et al. 2021), rather than the activity of the analysed enzyme (Nandekar et al. 2016; Nguyen et al. 2022). An alternative explanation could be intestinal cell autophagy which provides protection against the harmful effects of DON (Tang et al. 2015).

Our findings corroborate the results of an in vitro study analysing the influence of ZEN and DON on the same genes (Smith et al. 2017) in the same weeks of exposure. In both studies, the *m*RNA expression of *CYPs* decreased after 41 days of exposure (Fig. 3 DIII), which suggests that low mycotoxin doses are tolerated by pre-pubertal gilts.

Maybe the excess of enzymes is due to the fact of interplay or correlation between the expression of ERs, the level of expression of enzyme genes and the level (deficiency of endogenous oestrogens) of the entry of ZEN and its metabolites causing only what?—hyperoestrogenism or supraphysiological hormonal levels in pre-pubertal gilts. This means that in this situation there is no silencing only intensification of enzyme expression, which is confirmed by the results of our experiment, but only in the ZEN group, or possibly in the MIX group. In the other two there is silence. At the same time, there is an increased expression of ERs.

The *m*RNA expression of *GSTπ1* increased steadily only in the CON group (Fig. 4). In the remaining groups, *GSTπ1* expression was higher than in the CON group (Elofey et al. 2020), with the most pronounced decreasing trend in the MIX group. The *m*RNA expression of *GSTπ1* was highest in the ZEN group, and it slightly decreased in successive dates of exposure.

The data presented in Fig. 4 DI indicate that in the first date of the experiment, *GSTπ1* expression increased in all experimental groups relative to the CON group. The highest increase was noted in the ZEN group. In the remaining dates of exposure, *GSTπ1* expression was higher in ZEN and DON groups. Once again, the above can be attributed to the fact that DON, unlike other mycotoxins, is not biologically active in the first phase of biotransformation, and it is incapable of forming more toxic compounds (Elofey et al. 2020; Payros et al. 2016) or it undergoes microbiological transformation to DOM-1.

A comparison of our findings with the results reported by Gouze et al. (2006) and Wu et al. (2015) suggests that DON could be a substrate for glutathione S-transferase in the second phase of biotransformation or that DON could induce oxidative stress (You et al. 2021) in cells due to higher intracellular production of reactive oxygen species (ROS) (Sun et al. 2015). The above processes could have taken place in the MIX group where *GSTπ1* expression was fairly stable throughout the experiment (Fig. 4) and where the lowest expression values relative to the remaining groups were noted in the last two dates of exposure (Fig. 4 DII and DIII). The results noted in the MIX group suggest that an antagonistic interaction (Martins et al. 2018) occurred between the analysed mycotoxins or that the presence of both mycotoxins inhibits the catalytic activity of the analysed enzymes. Mycotoxin-induced disruptions in hepatic oestrogen clearance could gradually increase the accumulation of oestrogens in pre-pubertal gilts (El-Hefnawya et al. 2017).

In conclusion, the results of the study support the hypothesis that ZEN and DON, applied alone or in combination, are immunoreactive towards ER*α* and ER*β* and influence the expression of genes encoding liver enzymes that participate in the biotransformation and neutralisation of undesirable substances. Gene expression tended to decrease gradually during the experiment. The level of expression of the analysed genes may point to disruptions in specialised liver functions (Smith et al. 2017) and variable biological activity of the tested mycotoxins at different stages of biotransformation (Payros et al. 2016). This suggests that the biological activity of the parent compounds is related to their metabolic activity (Shimizu et al. 2021). Therefore, considering the effect exerted by mycotoxins on energy-intensive processes and their endogenous metabolism, it appears that the observed situation may lead to the activation of adaptation mechanisms (Gajęcka et al. 2017; Yoon et al. 2020). In addition, mycotoxin-induced disruptions in hepatic oestrogen clearance may gradually increase the accumulation of oestrogens in pre-pubertal gilts (El-Hefnawya et al. 2017), which increases the intensity of metabolic processes. The involvement of both mycotoxins in the expression of *ERs*, *CYP1A1* and *GSTπ1* has practical significance, but further research is required to determine their applicability for diagnostic purposes. However, the observed processes do not support the determination of pharmacokinetic parameters which could be used to optimize mycotoxin doses.

## Data Availability

The datasets generated during and/or analysed during the current study are available from the corresponding author on reasonable request.
